# Formation of High-Purity Indium Oxide Nanoparticles and Their Application to Sensitive Detection of Ammonia

**DOI:** 10.3390/s151229895

**Published:** 2015-12-17

**Authors:** Sanjeev K. Bhardwaj, Neha Bhardwaj, Manil Kukkar, Amit L. Sharma, Ki-Hyun Kim, Akash Deep

**Affiliations:** 1 Central Scientific Instruments Organisation (CSIR-CSIO), Sector 30 C, Chandigarh 160030, India; sanjeevbhardwaj1988@gmail.com (S.K.B.); nehavashisht1989@gmail.com (N.B.); er.manilkukkar@gmail.com (M.K.); amitsharma_csio@yahoo.co.in (A.L.S.); 2 Academy of Scientific and Innovative Research, CSIR-CSIO, Sector 30 C, Chandigarh 160030, India; 3 Department of Civil & Environmental Engineering, Hanyang University, 222 Wangsimni-Ro, Seoul 04763, Korea

**Keywords:** indium oxide nanoparticles, recovery, scrap, sensing, ammonia

## Abstract

High-purity In_2_O_3_ nanoparticles were recovered from scrap indium tin oxide substrates in a stepwise process involving acidic leaching, liquid-liquid extraction with a phosphine oxide extractant, and combustion of the organic phase. The morphological and structural parameters of the recovered nanoparticles were investigated to support the formation of the desired products. These In_2_O_3_ nanoparticles were used for sensitive sensing of ammonia gas using a four-probe electrode device. The proposed sensor offered very quick response time (around 10 s) and highly sensitive detection of ammonia (at a detection limit of 1 ppm).

## 1. Introduction

Indium oxide (In_2_O_3_) nanostructures have a direct bandgap of 3.55–3.75 eV with versatile applicability to selective sensors, solar cells, flat-panel displays, and photocatalytic devices [1,2,3,4]. The wide band gap of In_2_O_3_ suggests their potential applicability under room temperature conditions, like other wide band gap semiconductors, e.g., GaN, ZnO, TiO_2_, and diamond. A number of synthesis methods have been proposed to grow In_2_O_3_ nanostructures in different forms such as nanowires, nanowire arrays, nanocubes, octahedrons, hollow microspheres, and nanorod bundles (and spheres) [5]. For the synthesis of indium oxide nanostructures, the following techniques have commonly been employed: electrodeposition, chemical bath deposition, chemical vapour deposition, molecular beam epitaxy, hydrothermal, and sol-gel synthesis [6]. Chemical deposition is a simple and low cost method. However, wet chemical synthesis often requires a variety of controlling agents to adjust the shape of nanocrystals. The use of such additives (e.g., complexing agents, surfactants, and pH regulating agents) makes the synthesis methods complicated and costly. Sometimes those additives can also remain as impurities in the final product. Electrodeposition and oxidizing methods are used preferably to obtain indium oxide spheres [7]. The use of chemical vapor deposition and annealing of In(OH)_3_ precursor have been proposed as possible routes for the synthesis of In_2_O_3_ nanoparticles [8,9]. Hard and soft template-based synthesis of different In_2_O_3_ micro- and nanostructures has also been proposed by several researchers [10,11,12,13]. Use of polystyrene beads, polymethylmethacrylate, and carbon materials has been reported in hard template-based synthesis, whereas vesicles, microemulsion droplets, and micelles have been used in soft template-based synthesis. In the preparation of In_2_O_3_ particles, application of the former was preferred over the latter due to superiority in terms of size, shape, shell thickness, and surface morphology.

The application of In_2_O_3_ nanoparticles for sensing ammonia has been demonstrated by many researchers [14,15,16]. However, most of those reported sensors were designed to operate at elevated temperatures. In some cases, response times of 30 s or more have been reported [14,15]. Other studies have also addressed the possibility of sensing ammonia under room temperature conditions without providing detailed information [15]. Ti^4+^ ion doped In_2_O_3_ has also been proposed for such an application with the detection of NH_3_ in the 5–1000 ppm range [16].

Because indium is a rare element, its regeneration from scrap materials is important. Leaching has been proposed as an effective method to recover metallic components from scrap materials; however, it is difficult to obtain pure products using such an approach [17,18]. Liquid-liquid extraction is a popular hydrometallurgical technique that allows pre-concentration and purification of metals from leaching solutions [19,20,21]. In this research, we propose a new facile synthesis route for the formation of In_2_O_3_ nanoparticles from scrap indium tin oxide substrates based on an extraction-calcination route. The template-free method is capable of producing homogenously porous In_2_O_3_ nanoparticles. Compared to the above listed techniques, the process proposed herein is suitable for the enhanced recovery of the desired product from waste materials; it was proven to provide the *in-situ* separation of pure indium from other impurities (liquid-liquid extraction step). The utility of these In_2_O_3_ nanoparticles was demonstrated for sensitive sensing of ammonia gas by a four-probe sensor device. The synthesized In_2_O_3_ nanoparticles allowed sensitive (1 ppm) detection of ammonia.

## 2. Experimental

### 2.1. Materials

To build biosensing platform proposed in this research, scrap indium tin oxide (ITO) collected through the various optimization procedures tested in this study were used as the source of indium. The metal extractant “Cyanex 923” is a mixture of four alkyl phosphine oxides (R_3_P = O, R′R_2_P = O, R_2_R′P = O, and R_3_′P = O, where R and R′ represent *n*-octyl and *n*-hexyl hydrocarbon chains, respectively) marketed by the Cytec Canada Inc. (Paterson, NJ, USA) All other reagents and solvents used in this work were high-purity products from Sigma Aldrich/Merck (New Delhi, India).

### 2.2. Method

About 2 g of the scrap ITO substrates were cut in small pieces and then treated with 10 mL of 50% *aqua regia* solution (a 1:3 (*V/V*) mixture of HNO_3_ and HCl) for 1 h at 90 °C. The leaching solution (L1) was collected after this reaction. The conductivity of the remaining substrates was measured in order to verify the removal of the ITO layer. None the tested substrates showed any conductivity, indicating that the ITO layer was removed from the glass surface.

The leaching solution “L1” was diluted with deionized water to a volume four-times the original. The concentrations of the metals (indium, tin) in the solution were determined by inductively coupled plasma-mass spectrometry (ICP-MS, ELAN DRC-e, Perkin Elmer, Boston, MA, USA). The extraction of indium was then carried out in 0.05 mol/L toluene (Cyanex 923) solution. For this, 40 mL of the diluted “L1” solution was equilibrated with 10 mL of Cyanex 923 solution for 5 min at room temperature. The aqueous and organic layers were allowed to settle, and the organic phase (O1) was decanted. ICP-MS analysis of the aqueous phase was then carried out to determine the content of extracted metal.

For the preparation of In_2_O_3_ nanoparticles, the solution “O1” was transferred into a platinum crucible and then combusted in a furnace at 500 °C for 2 h. The solvent was evaporated to produce finely assembled In_2_O_3_ particles, which were characterized by field-emission scanning electron microscopy-energy dispersive X-ray spectroscopy (FE-SEM-EDX, 4300/SN Hitachi, Tokyo, Japan), transmission electron microscopy (TEM, JEOL, Tokyo, Japan), Fourier transform infrared spectroscopy (FTIR, iS10, Nicolet, Madison, WI, USA), and X-ray diffraction spectroscopy (D8 Advance, Bruker, Mumbai, India). Surface area was measured using an Autosorb 1C BET Surface Area & Pore Volume Analyzer Quantachrome (Jacksonville, FL, USA). The particle size distribution analysis was carried out with a Zetasizer Nano ZS from Malvern (Worcestershire, UK).

The ammonia sensing device was fabricated by drop-casting the In_2_O_3_ suspension onto a four-probe gold electrode. The above suspension was prepared by treating 50 mg of the prepared In_2_O_3_ nanoparticulates with a 20 mL mixture of ethanol and chloroform (1:1). The mixture was sonicated for 30 min and then left for 12 h. Large particles were separated by centrifugation at 1000 rpm. This treatment was repeated for three times to obtain a well dispersed transparent colloidal solution for drop casting the nanoparticle layer.

**Figure 1:**
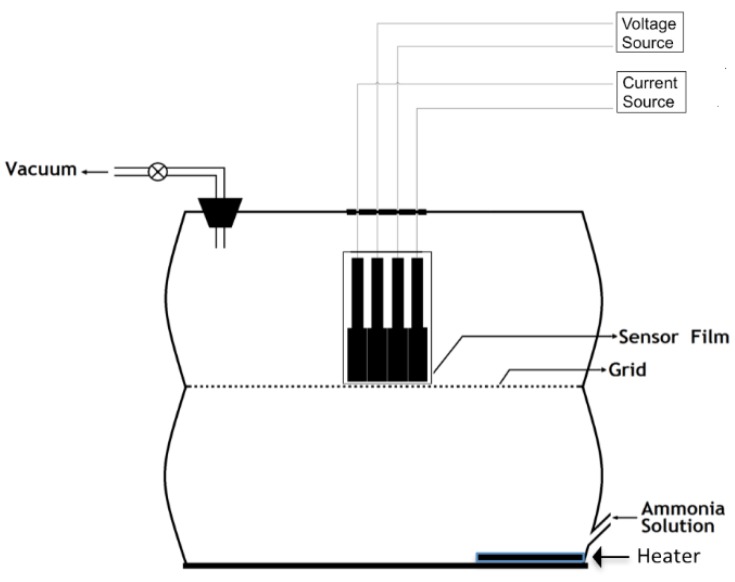
Schematic of the ammonia sensing setup.

The In_2_O_3_ drop-cased electrode was allowed to dry and then tested for detection of ammonia. The schematic set-up of the gas sensing chamber is shown in Figure 1. Suitably diluted ammonia solutions were introduced in the sensing chamber, and the gas was dispersed by heating so as to obtain effective gas concentrations in the range of 0.1–100 ppm. These concentrations were determined by first preparing ammonia solutions of known molarity and considering their maximum diffusion inside the gas chamber with a volume of 5.7 L. The sensitivity “S” of the sensor was estimated according to the following equation:

*S = R_a_/R_g_*

where *R_a_* is the resistance of the sensor in air and *R_g_* is the resistance after introducing ammonia into the test chamber. The response of the sensor was investigated using a constant current source and an electrometer (Keithley, Bangalore, India).

## 3. Results and Discussion

### 3.1. Solution Compositions

**Figure 2:**
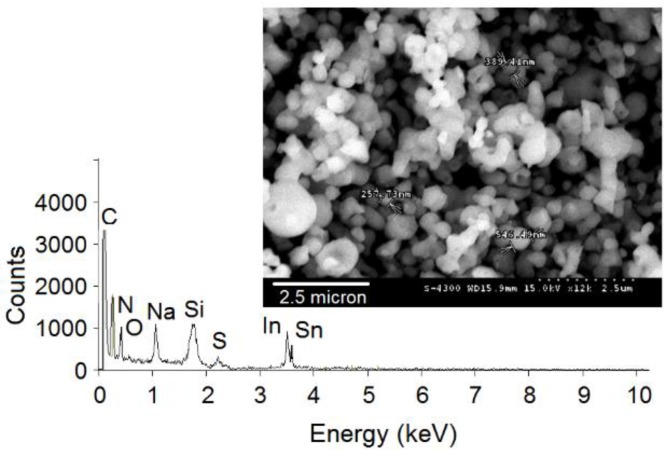
FE-SEM and EDX analysis of the solution obtained after the leaching step on scrap ITO substrates.

The ICP-MS analysis of solution “L1” before extraction with Cyanex 923 showed the following: 15 mg/L of indium and 2 mg/L of tin. The literature reports that acidic leaching of ITO is useful to solubilize indium, while the leaching of tin remains is only about 10% [21]. Because the target substrates were scraps collected during optimization studies for the development of biosensors, the presence of some components like carbon, nitrogen, and sulfur in the leaching solution is expected. For complete analysis, a fraction of the “L1” solution was completely evaporated on a silicon wafer and analyzed using an FE-SEM/EDX, as shown in Figure 2. The presence of all the expected components (*i.e.*, indium, tin, carbon, nitrogen, and sulfur) is confirmed in this microstructure. The results of ICP-MS analysis after extraction with Cyanex 923 were as follows: indium, 0.1 mg/L and tin, 2 mg/L. This suggests that indium was almost completely transferred to the organic phase with negligible extraction of tin. This particular study clearly highlights the selective extraction of indium by Cyanex 923 solution. The extraction of metal ions with Cyanex 923 takes place via a solvation process that depends upon the acidity in the aqueous phase. The quantitative extraction of In(III) from dilute to moderate HCl solutions can be attributed to the formation of extractable InCl_3_ species. As tin forms neutral or cationic chloro-species only in concentrated HCl conditions, its extraction was negligible under the present aqueous phase conditions.

The results of these studies confirm the effectiveness of the proposed leaching-extraction technique for obtaining pure indium solution from scrap ITO targets. The suggested method can also be applied to other types of waste materials. The proposed process involves the solvent extraction separation of indium from a leaching solution. Hence, a similar strategy can also be applied to other waste materials.

### 3.2. Characterization of In_2_O_3_ Nanoparticles

The leaching, extraction, and combustion processes can be explained by the following chemical equations:

$$In_{x}Sn_{y}O_{z}+HCl/HNO_{3}\overset{Leaching\;at\;90\;℃}{\to }\;InCl_{3}+\;SnCl_{2}+Acid/H_{2}O$$

$${\left(InCl_{3}+SnCl_{2}+Acid/H_{2}O\right)}_{aqueous}+\;{\left(Cyanex\;923\right)}_{organic}\to \;{\left(InCl_{3}.2Cyanex\;923\right)}_{organic}+\;{\left(SnCl_{2}+Acid/H_{2}O\right)}_{aqueous}$$

$${\left(InCl_{3}.2Cyanex\;923\right)}_{organic}\overset{Combustion,\;\;\;500\;℃}{\to }\;In_{2}O_{3}$$

**Figure 3:**
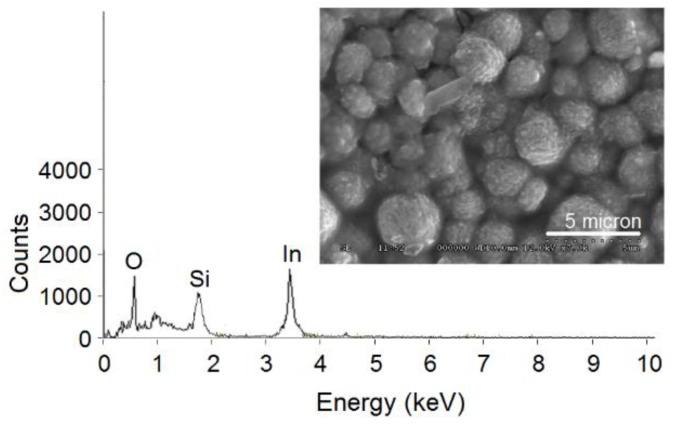
FE-SEM and EDX analyses of the synthesized In_2_O_3_ nanoparticles.

**Figure 4:**
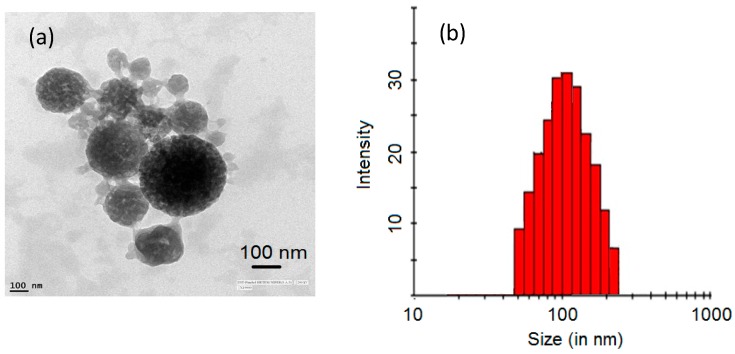
(**a**) TEM analysis and (**b**) Particle size distribution (DLS) of the synthesized In_2_O_3_ nanoparticles.

In the given composition of the leaching reagent, it can be assumed that indium should primarily be dissolved as InCl_3_. The extraction of indium chloro-complex by Cyanex 923 has been described in the literature through a solvation mechanism [22]. As discussed in the previous section, the extraction of indium from the leaching solution “L1” was selective towards tin. Combustion of the indium-Cyanex 923 complex led to the formation of porous nanoparticles of In_2_O_3_, as evidenced by FE-SEM and TEM investigations (Figure 3 and Figure 4a, respectively). The obtained product and its EDX analysis confirm the formation of pure homogeneous nanoparticles of In_2_O_3_ free from other impurities. The particle size distribution analysis of the In_2_O_3_ dispersion is shown in Figure 4b. A polydispersity index of 0.24 suggests that the In_2_O_3_ dispersant should contain homogenous particles. The average particle diameter was estimated as 100 nm.

The morphology of these particles indicates the porous nature of the nanoparticles. The estimated Langmuir surface area of the In_2_O_3_ nanoparticles averaged as 211 m^2^ g^−1^. The purity of the synthesized product has also been cross-checked with ICP-MS analysis. A digested sample of In_2_O_3_ was found to be free from other elements, namely, Sn, Fe, Al, Cr, Na, Cu, and Cr.

**Figure 5:**
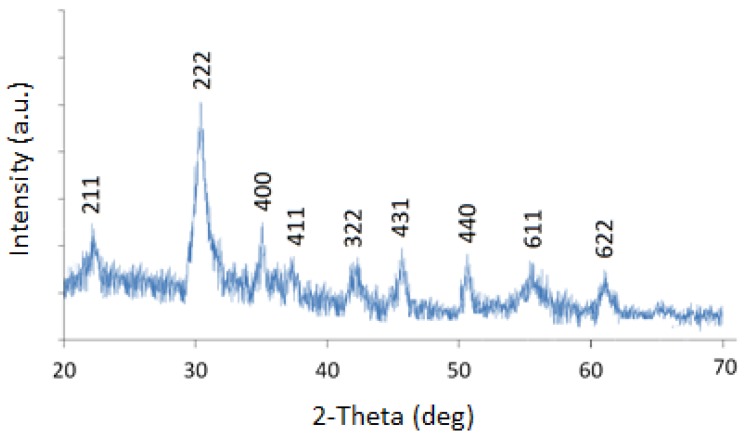
XRD analysis of the synthesized In_2_O_3_ nanoparticles.

XRD analysis of the sample is shown in Figure 5. The presence of strong and sharp diffraction peaks confirms synthesized product as a crystalline material.

**Figure 6:**
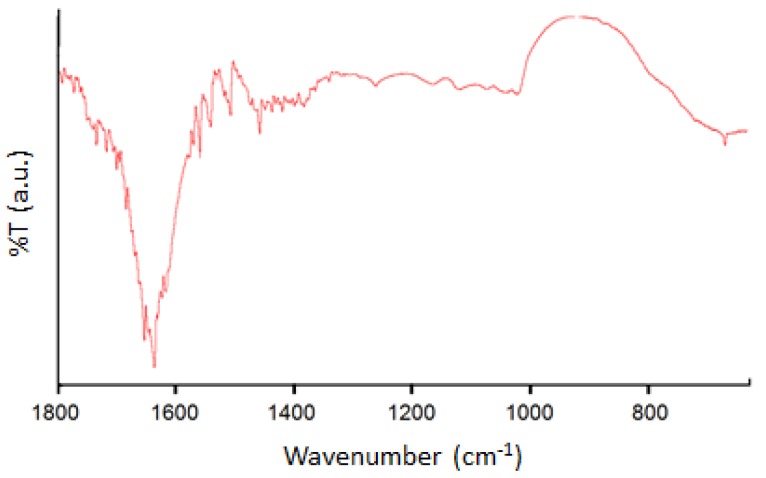
FTIR analysis of the synthesized In_2_O_3_ nanoparticles.

All the diffraction peaks can be indexed to body centered cubic (bcc) structured In_2_O_3_. The XRD data are in agreement with the standard data from JCPDS card No. 65-3170 as well as results of previous studies [14,23,24]. There were no impurities phases present. Figure 6 shows the FTIR spectra of the recovered In_2_O_3_ nanoparticles. The thermal treatment of the complex produced pure In_2_O_3_ nanoparticles. The bands around 1630 and 1050 cm^−1^ are attributed to the absorption of hydroxyls and absorption of C-O vibration, respectively. Broad absorptions around 600 cm^−1^ are attributed to the metal oxide vibrations. The results of the FTIR analysis were comparable to those of the earlier reports, e.g., [25].

### 3.3. Use of In_2_O_3_ Nanoparticles for Ammonia Sensing

**Figure 7:**
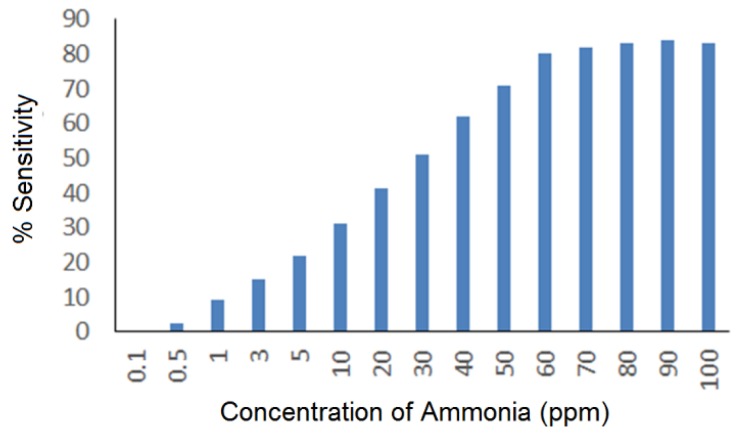
Sensitivity of the In_2_O_3_ nanoparticle sensor at different concentrations of ammonia.

**Figure 8:**
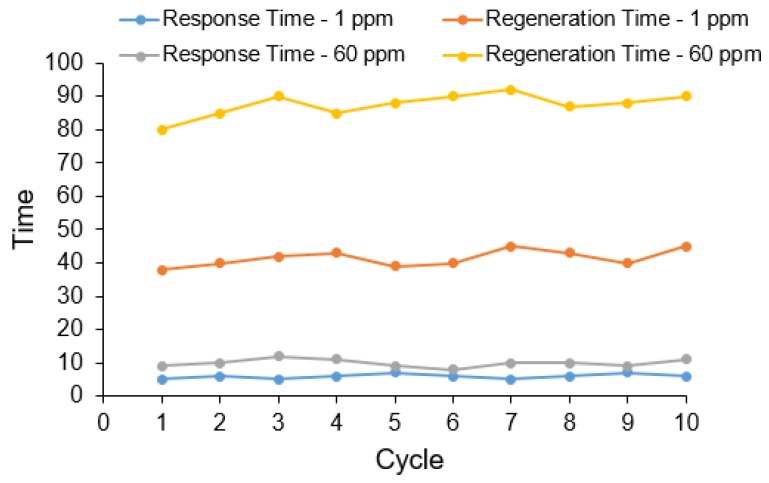
Response and recovery of the In_2_O_3_ nanoparticle sensor at low (A: 1 ppm) and high concentrations of ammonia (B: 60 ppm) during successive cycles.

The results obtained from the application of recovered In_2_O_3_ nanoparticles for the sensing of ammonia are shown in Figure 7 and Figure 8.

As shown, the sensor can very sensitively detect the presence of NH_3_ molecules in a linear detection range of 1 to 60 ppm. Exposure of the sensor to higher concentrations led to saturation. The investigations on the response time showed fast response of less than 10 s for 1 ppm, while it is slightly extended above 10 s for 60 ppm. The recovery times (Figure 8) measured at two different concentrations (1 and 60 ppm) were around 50 and 100 s, respectively. Ammonia behaved as a reducing gas for the In_2_O_3_ sensor. It is known that In_2_O_3_ in a slightly non-stoichiometric form contains a large number of localized centers with trapped electrons, which explains the observed sensitivity in the sensor’s response at room temperature. This response behavior indicates the possibility of many surface sites suitable for reaction with ammonia. A large proportion of the sensitivity resistance of the sensor is due to surface reactions. Upon exposure to air, oxygen species are adsorbed on the surface of a metal oxide semiconductor gas sensor. These oxygen species then undergo ionization by electrons from the material’s conduction band to form such species as O^2−^. The resistance of In_2_O_3_ semiconducting material is expected to increase in the presence of air due to relatively low concentration of free electrons in the conduction band. The presence of reducing gas (NH_3_) initiates the surface reaction between the oxygen species and analyte gas, causing the release of electrons trapped in the ionized oxygen species back into the conduction band, which ultimately lowers the resistance. This result is typical behavior for indium oxide as an n-type semiconductor. The porous structure of In_2_O_3_ is an important reason for the sensitivity of the sensor. Porosity of the surfaces helps in absorbing more oxygen to generate more ionized oxygen species. The increased surface-to-volume ratio of the porous nanostructure should increase the absorption and ionization of oxygen. The sensing performance of In_2_O_3_ obtained at two different calcination temperatures (*i.e.*, 500 and 700 °C) is summarized in Table 1. It indicated that the performance of the above two sets of the particles was not much different from each other.

**Table 1:** Relative performance of In_2_O_3_ for sensing ammonia at two different calcination temperatures between 500 and 700 °C.

Concentration of NH_3_	Parameters Observed for Material Calcined at 500 °C			Parameters Observed for Material Calcined at 700 °C		
	Sensitivity	Response Time	Recovery Time	Sensitivity	Response Time	Recovery Time
5 ppm	22 ± 2%	10 ± 4	50 ± 10	25 ± 2%	8 ± 4	40 ± 6
60 ppm	78 ± 3%	10 ± 4	100 ± 10	85 ± 4%	10 ± 4	85 ± 10

The chemiresistive sensors are potent enough to develop fast, inexpensive, and portable devices for sensing various gases. However, their sensitivity toward humidity is often found as a limiting factor in acquiring the reliable and reproducible results. To resolve this problem, the sensor can be designed with a reference line for blank controls as desired. As checked in the lab, the sensitivity of the sensor proposed herein toward 5 ppm ammonia/50% relative humidity was around 50:1. It might be pertinent to discuss here that various sensor designs have been proposed in the literature to counter this humidity-related problem. The interfering effect of humidity was tackled by virtue of the difference in thermal conductivity between air and water vapor. Application of standard passivation layer of the CMOS chip as the humidity sensing element, subtraction circuit, and self-calibrated humidity sensor may also be used to develop a complete sensing system [26]. As another approach to compensate humidity, a separate in-chamber humidity sensor was employed [27]. The data were successfully processed for post feature extraction compensation of humidity to accurately adjust bias in the measured humidity.

The attainment of required selectivity is another issue for the gas sensing devices. One of the approaches to handle this situation is to add computational hardware/software for the pattern recognition analysis of various output sensing signals [28]. Recently, some researchers have proposed the design of sensors that can supply an assortment of independent features each of which responds to the various analyte gases. The collective output of these features could be treated via simple pattern recognition methods against the complicated mixtures [29]. The selective detection of gases and volatile organic compounds can also be achieved by considering multiple independent parameters of a sensor, e.g., voltage threshold, hole mobility, subthreshold swing, *etc.* as input for artificial neural network which, in turn, can be trained in its ensemble to make the targeted detection.

## 4. Conclusions

For the first time, the recycling of used indium tin oxide substrate into conducting indium oxide nanoparticles is described. Pure (>98%) indium solution was retrieved from the leaching solution of scrap ITO targets using Cyanex 923 extractant. This solution was then subjected to combustion in order to produce nanoparticles. The synthesized In_2_O_3_ nanoparticles, when tested by various instrumental methodologies, were shown to be highly pure and useful for detection of ammonia gas. The proposed strategy can also be extended to other waste materials containing indium for its efficient recycling. As demonstrated in this study, the recovered product can be used as a gas sensing material in addition to other potential applications.
